# Genotyping of polyploid plants using quantitative PCR: application in the breeding of white-fleshed triploid loquats (*Eriobotrya japonica*)

**DOI:** 10.1186/s13007-021-00792-9

**Published:** 2021-09-03

**Authors:** Haiyan Wang, Jiangbo Dang, Di Wu, Zhongyi Xie, Shuang Yan, Jingnan Luo, Qigao Guo, Guolu Liang

**Affiliations:** 1 Key Laboratory of Horticulture Science for Southern Mountains Regions of Ministry of Education, College of Horticulture and Landscape Architecture, Southwest University, Beibei, Chongqing, 400715 China; 2 State Cultivation Base of Crop Stress Biology for Southern Mountainous Land of Southwest University, Academy of Agricultural Sciences of Southwest University, Beibei, Chongqing, 400715 China

**Keywords:** qPCR genotyping, Polyploid, Flesh color, Allele dosage, Polyploid breeding, Loquat

## Abstract

**Background:**

Ploidy manipulation is effective in seedless loquat breeding, in which flesh color is a key agronomic and economic trait. Few techniques are currently available for detecting the genotypes of polyploids in plants, but this ability is essential for most genetic research and molecular breeding.

**Results:**

We developed a system for genotyping by quantitative PCR (qPCR) that allowed flesh color genotyping in multiple tetraploid and triploid loquat varieties (lines). The analysis of 13 different ratios of DNA mixtures between two homozygous diploids (AA and aa) showed that the proportion of allele A has a high correlation (R^2^ = 0.9992) with parameter b [b = a_1_/(a_1_ + a_2_)], which is derived from the two normalized allele signals (a_1_ and a_2_) provided by qPCR. Cluster analysis and variance analysis from simulating triploid and tetraploid hybrids provided completely correct allelic configurations. Four genotypes (AAA, AAa, Aaa, aaa) were found in triploid loquats, and four (AAAA, AAAa, AAaa, Aaaa; absence of aaaa homozygotes) were found in tetraploid loquats. DNA markers analysis showed that the segregation of flesh color in all F_1_ hybrids conformed to Mendel's law. When tetraploid B431 was the female parent, more white-fleshed triploids occurred among the progeny.

**Conclusions:**

qPCR can detect the flesh color genotypes of loquat polyploids and provides an alternative method for analyzing polyploid genotype and breeding, dose effects and allele-specific expression.

**Supplementary Information:**

The online version contains supplementary material available at 10.1186/s13007-021-00792-9.

## Background

Polyploidization is an important driving force in the evolution of eukaryotes [1]. Ancient whole-genome duplication (WGD) events resulting in polyploidy in various plants have promoted the innovative development of plants and provided a basis for plant diversification [2, 3]. Polyploidy resulting from WGD can overcome self-incompatibility and interspecific hybrid sterility, which is beneficial for the production of germplasm resources with research and application value [4, 5]. The number of genomes is an important factor affecting the fertility of plants; an even number of genomes results in higher fertility, while an odd number of genomes may result in sterility [6]. Changes in plant ploidy levels will lead to many phenotypic changes that may deviate from Mendel’s law of inheritance [7]. These fertility and trait changes brought about by the broad changes in ploidy provide abundant raw materials for the breeding system, allowing researchers to develop different breeding plans according to different breeding goals. At present, polyploid breeding is carried out in many crops and horticultural plants, such as wheat [8], watermelon [9], banana [10] and citrus [11].

Loquat (*Eriobotrya japonica* (Thunb.) Lindl.) is a subtropical evergreen fruit tree with good flavor and rich nutritional value that is widely cultivated worldwide [12]. Modern loquat cultivars have originated from a single domesticated event, and they are no significant different from wild species because of the short history of domestication [13]. Recent research suggests that the apple tribe (apple, pear and loquat) shared a WGD from a common ancestor with nine chromosomes [14], and found the frequency of large-scale fragment rearrangements [15]. The loquat chromosomes LG1/LG2, LG7/LG8 and LG11/LG13 showed high homologous relationships, but no high density and equally distributed genetic linkage map has been constructed [15, 16].

On account of the low edible rate caused by the large and multiple of seeds, less seeded or seedless have always been the ideal traits of loquat [17]. Existing triploid loquats are mostly selected from diploid seedlings, which is a time-consuming, labor-consuming and inefficient [18]. Hybrids of tetraploid and diploid plants are triploid and show strong heterosis [19]. The breeding efficiency achieved via this method is significantly higher than that of other methods [11, 20].

Flesh color is one of the core indicators of fruit appearance quality. Loquats can be divided into red-fleshed and white-fleshed loquats, and white-fleshed loquats are the main breeding goal of current breeders because they are more delicate, sweet and economically beneficial [21–23]. However, the early selection of fruit trees with a long juvenile period is more difficult, and marker-assisted selection (MAS) is currently the best solution to this issue [24]. The carotenoid content is the determinative factor in loquat flesh color, and which is controlled by *EjPSY2A* and *EjPSY2A*^*d*^ [25]. The molecular markers corresponding to the *EjPSY2A*^*d*^ deletion fragment can be used to quickly identify the flesh color types of loquat. Previous studies have shown that *EjPSY2A* and *EjPSY2A*^*d*^ are a pair of alleles in diploid loquat [23], *EjPSY2A* is the dominant allele responsible for red flesh (denoted by A), and *EjPSY2A*^*d*^ is the recessive allele responsible for white flesh (denoted by a); the red-fleshed genotype is AA or Aa, and the white-fleshed genotype is aa.

However, the complex nature of polyploids makes molecular genetics research more difficult, such as larger number of genotypic classes, possibility of multivalent pairing, poor knowledge of chromosome behavior during meiosis, and the change in chromosome copy number [26]. For the flesh color, red-fleshed tetraploid loquats present three genotypes: AAAa, AAaa and Aaaa, and red-fleshed triploid loquats present two genotypes: AAa and Aaa [27]. The segregation of flesh color differs among the hybrids of different heterozygotes. If the alleles have additive effects, or by creating more complex interactions between loci or alleles, it is necessary to accurately identify heterozygous genotypes because their phenotypes may be different [28–30].

Most of the existing genotyping techniques only detect the existence of alleles and do not provide their relative ratios [31–33]. Quantitative genotyping strategies can determine the relative ratios of alleles; these methods include genotyping by sequencing (GBS) [34, 35], quantitative fluorescent polymerase chain reaction (QF-PCR) [36, 37], high-resolution digestion (HRM) [38], competitive allele-specific PCR [39]. These methods are usually expensive and complicated, which greatly limits their application scope. Hence, for polyploid genotyping, a more economical and convenient technique must be found. Quantitative real-time polymerase chain reaction (qPCR) is often used to detect gene expression [40], copy number variation [41] and aneuploidy [42], and has high application potential for genotyping and other analyses.

In this study, loquats of different ploidies were used as experimental materials, and the flesh color genotypes of polyploid loquat were determined by using qPCR. The DNA pool mixed with two homozygous diploids (AA and aa) at 13 different ratios was used to analyze the feasibility of qPCR genotyping. The clarification of tetraploid loquat genotypes facilitates research on hereditary regularities in the progenies of polyploid hybridizations. qPCR genotyping can detect the genotypes of important traits in polyploid plants without whole genome sequencing data, which provides an alternative for plant genetic improvement and genetic analysis.

## Results

### Flesh color types of loquats of different ploidies

**Fig. 1:**
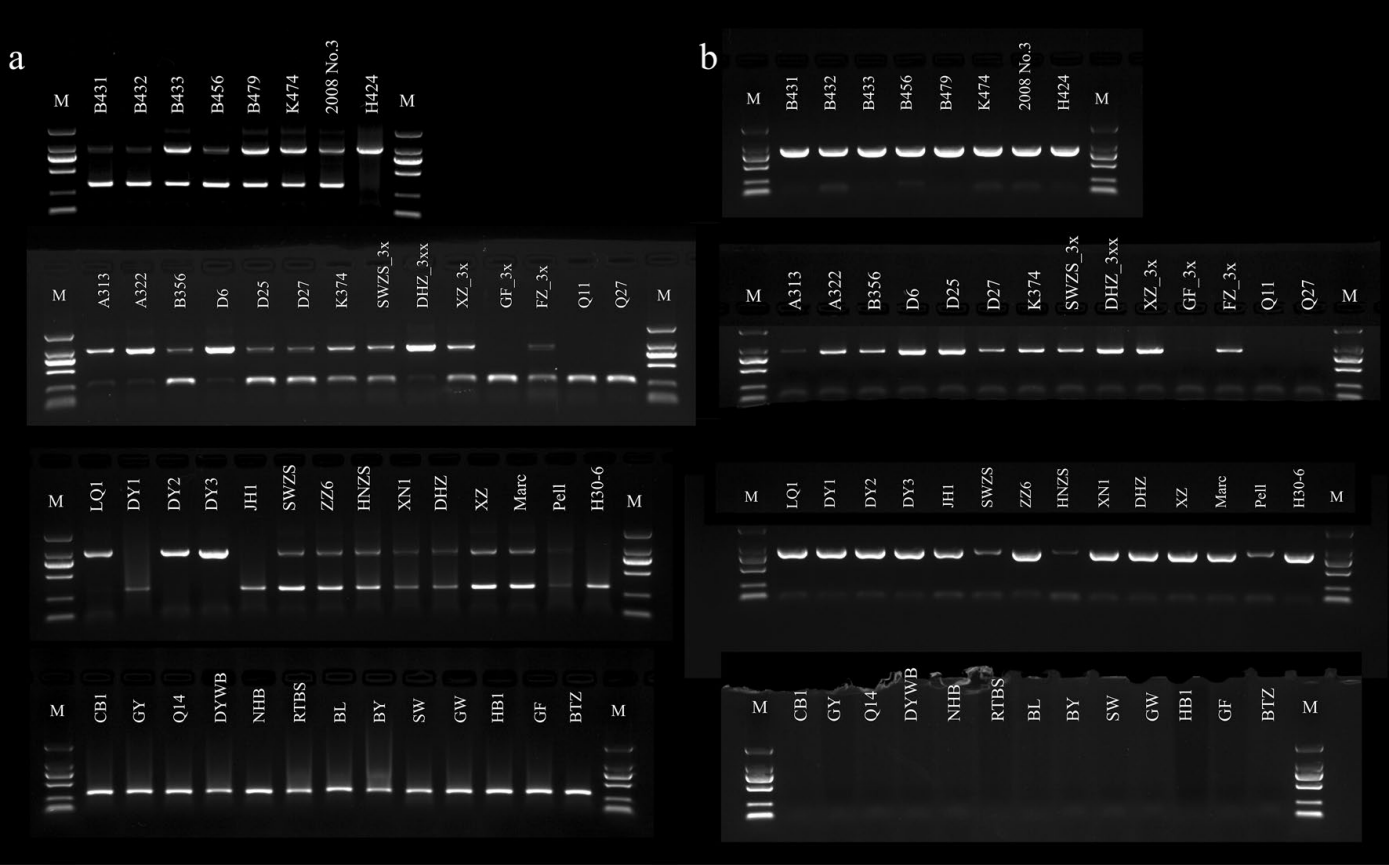
Electrophoretogram of flesh color-specific markers in polyploid loquats. **a** The specific marker of flesh color. **b **The improved specific marker of flesh color. The figures from top to bottom are for tetraploid, triploid, red-fleshed diploid, and white-fleshed diploid loquats. The marker is a DL 2000 DNA marker

Among the tetraploid loquats, H424 was the only red-fleshed homozygote, and the other seven lines were red-fleshed heterozygotes. Among the triploid loquats, there were four red-fleshed homozygotes, seven red-fleshed heterozygotes, and three white-fleshed homozygotes. The diploid loquat group included three red-fleshed homozygotes, eleven red-fleshed heterozygotes, and thirteen white-fleshed homozygotes (Fig. 1a). The flesh color-specific marker was not easy to observe in some red-fleshed heterozygous materials (‘DY1’, ‘JH1’, ‘Pell’ and ‘H30-6’), which affected the accuracy of the identification of unknown materials. The identification accuracy rate for the improved flesh color markers was 100%, the bands were clear and easy to identify, and the markers presented high application value (Fig. 1b).

### Effectiveness of the qPCR genotyping system

**Fig. 2:**
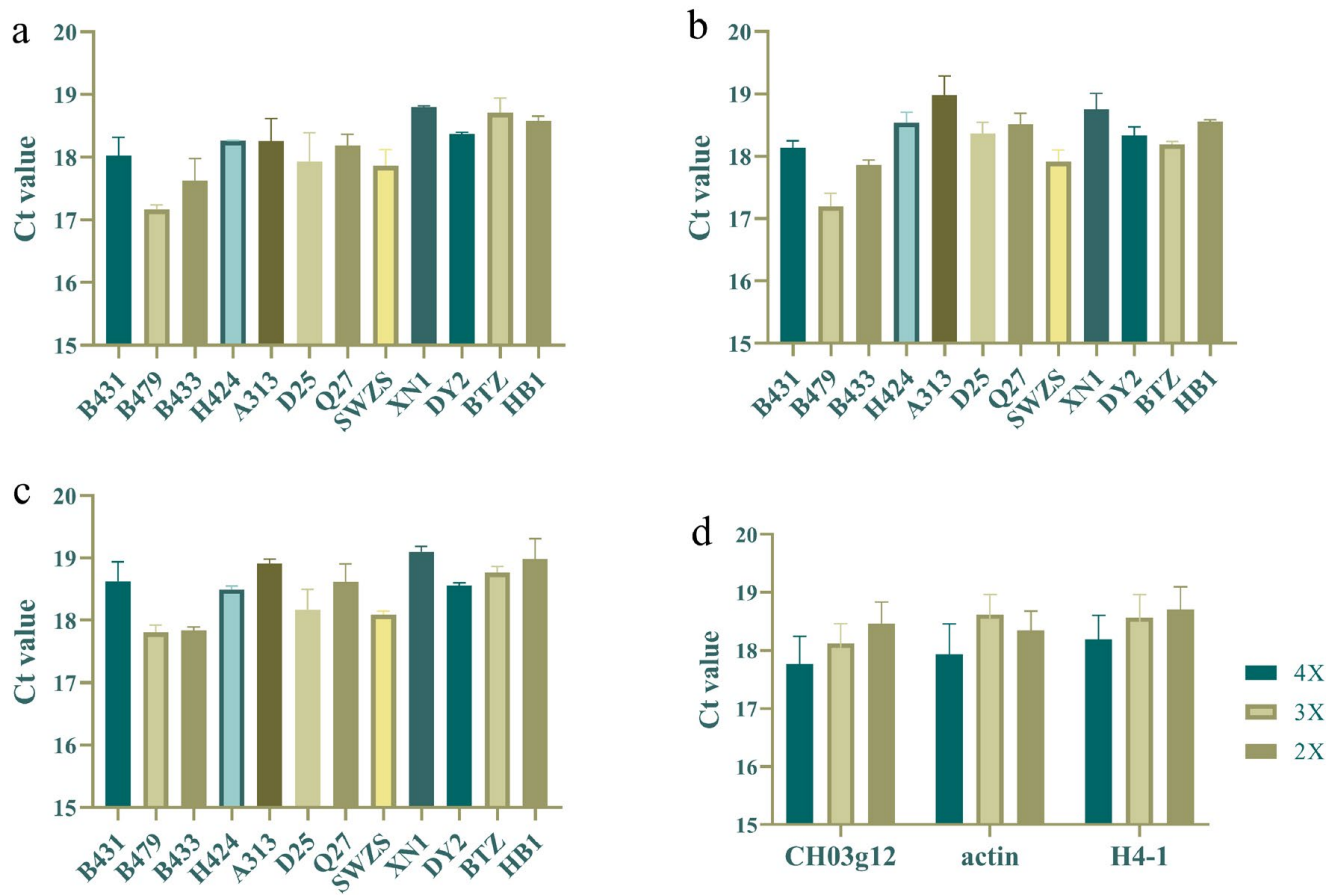
The Ct values of the reference sequence of the qPCR genotyping system in loquats of different ploidies. **a**–**c** Histograms of the Ct values of Ch03g12, *actin* and *H4-1* in loquats of different ploidies. **d** Data comparison summary of the preceding three panels

**Fig. 3:**
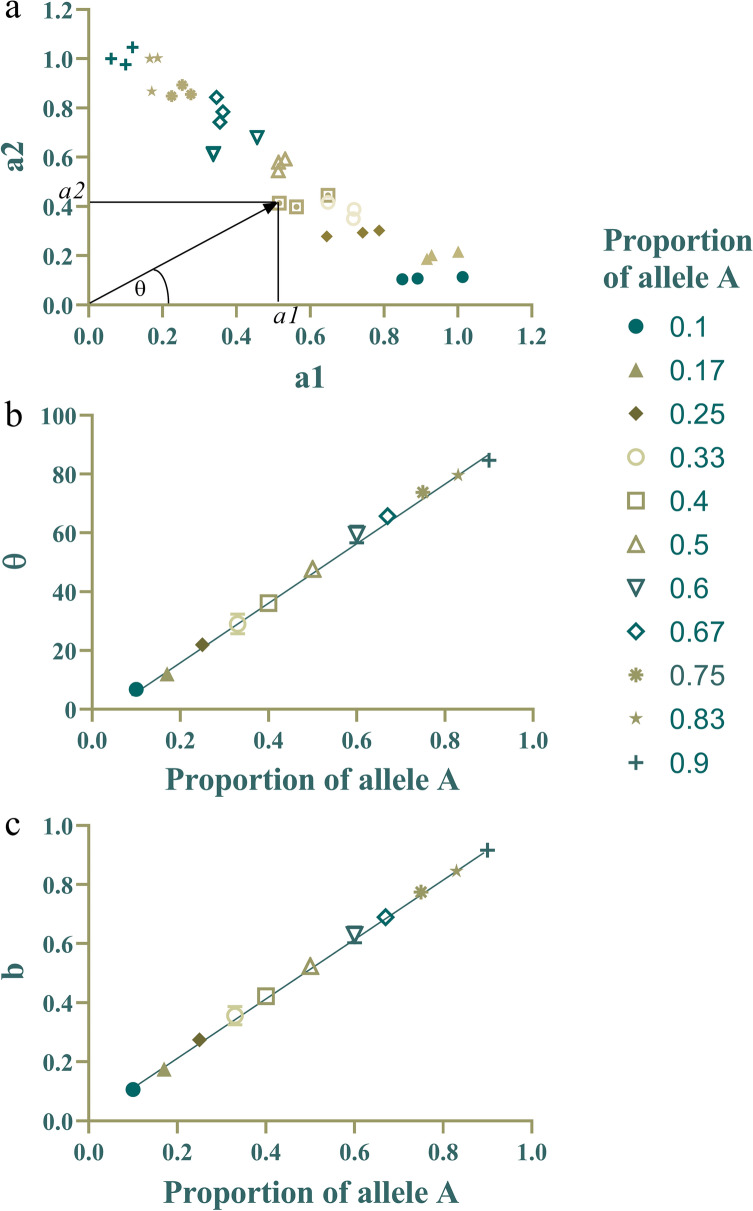
Correlation analysis between the proportion of allele A and the relative dose of allele A. **a** Distribution diagram of the relative copy number of allele A (a_1_) and the relative copy number of allele a (a_2_). **b** Correlation analysis between the proportion of allele A and angle θ [θ = tan^−1^(a_2_/a_1_)]. **c** Correlation analysis between the allele A proportion and parameter b [b = a_2_/(a_2_ + a_1_)]

The reference sequence was CH03g12, which presented stable qPCR results in loquat genomes of different ploidies (Fig. 2). The coefficients of variation (CVs) of the Ct values in the three reference sequences for the loquats of each ploidy were all less than 3%. CH03g12 showed a good linear relationship with ploidy. The specific primers q2A and q2A/2Ad presented high specificity, and their amplification efficiency was basically the same (Additional file 2: Fig. S2). To determine the significance of the genotyping system results, we used the W-mix and R-mix to prepare a DNA library in which the allele A occurred in 13 different proportions. Three repetitions were performed to analyze the correlation between the proportion of allele A and the relative dose of allele A, as shown in Fig. 3. The relative copy number of allele A (a_1)_ and the relative copy number of allele a (a_2_) were used to calculate the θ angle [θ = tan^−1^ (a_2/_a_1_)] and parameter b [b = a_2_/(a_1_ + a_2_)]. The correlation coefficient (R^2^) between the proportion of allele A and the θ angle was 0.9970, and that for parameter b was 0.9992, proving that the proportion of allele A showed a high correlation with the relative dose of allele A. These data indicate that the system can be used to determine the flesh color genotypes of polyploid loquats. Because the correlation coefficient of parameter b was higher, parameter b was used in subsequent experiments.

### Cluster analysis and ANOVA of simulated triploid and tetraploid allele doses

**Fig. 4:**
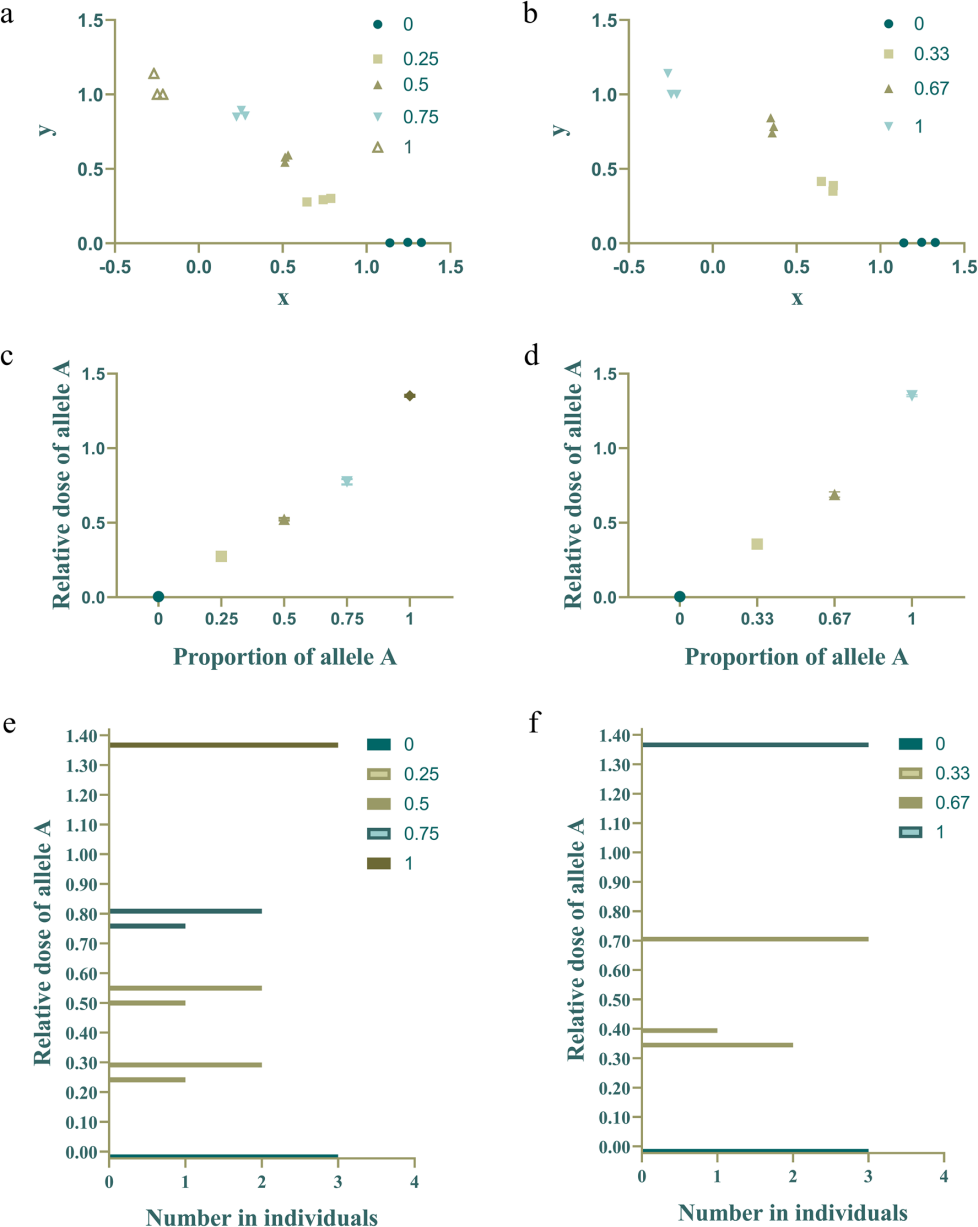
Cluster analysis and ANOVA of simulated triploid and tetraploid allele doses. **a**, **b** Showed the cluster analysis results of simulated tetraploid and triploid. **c**, **d** Showed the ANOVA results of simulated tetraploids and triploids. **e**, **f** Are the frequency histograms of the relative doses of allele A for simulated tetraploids and triploids.

A cluster analysis and ANOVA of the relative allele A signals (b parameter) in the simulated triploid and tetraploid populations were performed (Fig. 4). The simulated triploid and tetraploid groups included two and three heterozygotes, respectively. As ploidy increases, more heterozygotes may occur. The ANOVA showed that the proportions of simulated A alleles were accurately distinguished. The F value of tetraploids was 4751, P < 0.0001; the F value of triploids was 2954, P < 0.0001. The two simulation experiments were clustered via the farthest-neighbor method. All of the results were categorized into the correct classifications.

### qPCR genotyping of loquats of different ploidies with different flesh colors

**Fig. 5:**
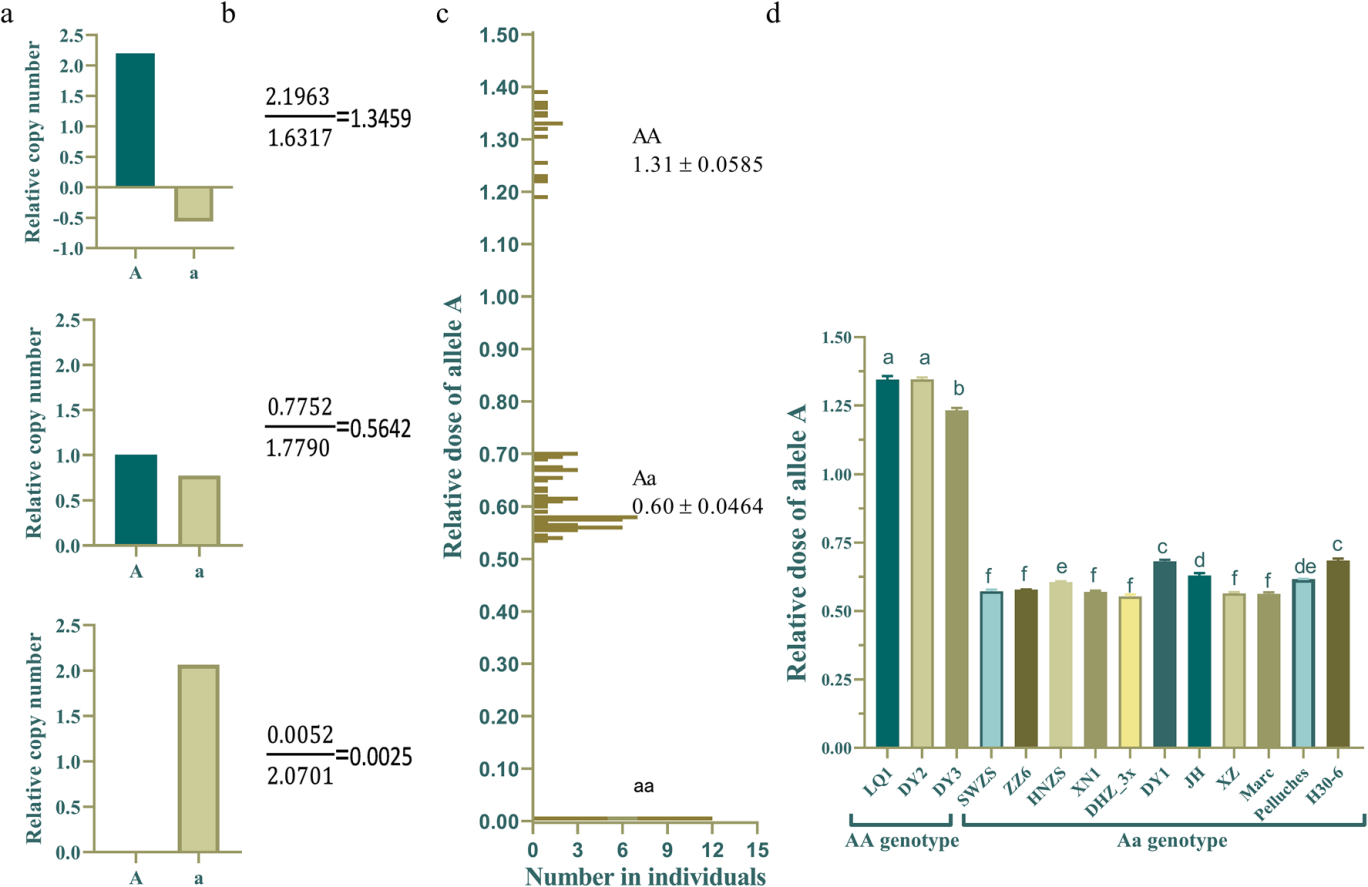
Genotyping results of diploid loquats. **a** Histogram of different genotypes of diploid loquats. **b** Relative doses of allele A in different genotypes. **c** Frequency histogram of different genotypes of diploid loquats. **d** Histogram of the relative doses of allele A in red-fleshed diploids

Diploid loquat was used for the verification of the qPCR genotyping system, and the results showed that the system could accurately determine the flesh color genotype. For the red-fleshed homozygotes (AA), parameter b was 1.31 ± 0.0585; for the red-fleshed heterozygotes (Aa), parameter b was 0.60 ± 0.0464; and for the white-fleshed homozygotes (aa), parameter b was 0.00 ± 0.0012. These results tended toward the expected values (AA = 1, Aa = 0.5, aa = 0) and proved the accuracy and stability of qPCR genotyping system (Fig. 5). Unexpectedly, the parameter b values of the four lines ‘DY1’, ‘JH1’, ‘Pell’ and H30-6 were slightly higher than those of the other lines, and this difference was significant (reference, ‘ZZ6’). However, the difference was not sufficient to affect the determination of the genotype. These materials were also included several materials that are difficult to analyze with flesh color-specific markers. It is speculated that gene copy number variation may exist among them, the specific reasons for this issue need to be further studied.

**Fig. 6:**
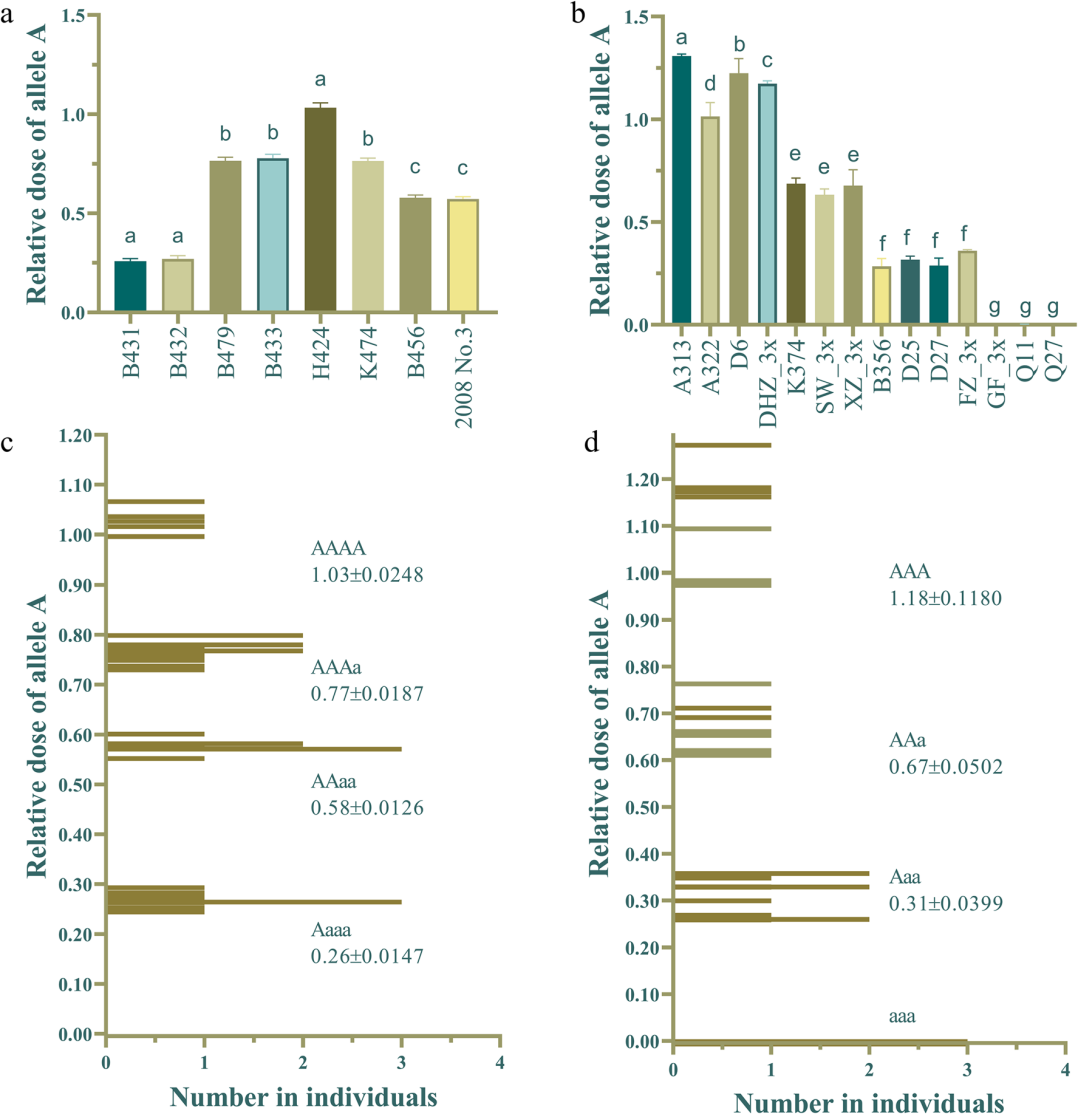
Genotyping results of triploid and tetraploid loquats. **a**, **b** Histogram of the relative doses of allele A in tetraploid and triploid loquats. **c**, **d** Frequency histogram of the relative doses of allele A in tetraploid and triploid loquats

The qPCR genotyping results for triploids and tetraploids are shown in Table 1. Among the triploids, A313, A322, D6 and DHZ_3x were red-fleshed AAA homozygote, and their parameter b was 1.18 ± 0.1180; K374, SW_3x and XZ_3x were AAa heterozygote, and their parameter b was 0.67 ± 0.0502; B356, D25, D27 and FZ_3x were Aaa heterozygote, and their parameter b was 0.31 ± 0.0399; the remaining lines were white-fleshed aaa homozygote, and their parameter b was 0.00 ± 0.0010 (Fig. 5). Among the tetraploid, H424 was only red-fleshed homozygote (AAAA), and its parameter b was 1.03 ± 0.0248; B431 and B432 were Aaaa heterozygote, and their parameter b was 0.26 ± 0.0147; B456 and 2008 No. 3 were AAaa heterozygote, and their parameter b was 0.58 ± 0.0126; B479, B433 and K474 were AAAa heterozygote, and their parameter b was 0.77 ± 0.0187 (Fig. 6).

**Table 1:** Genotyping results of polyploid loquats

Variety (lines)	Ploidy	Parameter b	Specific DNA marker of flesh color	Genotype
A313	2n = 3x = 51	1.30 ± 0.0068	Red homozygotic type	AAA
A322	2n = 3x = 51	1.01 ± 0.0682	Red homozygotic type	AAA
D6	2n = 3x = 51	1.22 ± 0.0508	Red homozygotic type	AAA
DHZ_3x	2n = 3x = 51	1.17 ± 0.0101	Red homozygotic type	AAA
K374	2n = 3x = 51	0.69 ± 0.0269	Red heterozygotic type	AAa
SW_3x	2n = 3x = 51	0.63 ± 0.0290	Red heterozygotic type	AAa
XZ_3x	2n = 3x = 51	0.68 ± 0.0776	Red heterozygotic type	AAa
B356	2n = 3x = 51	0.28 ± 0.0388	Red heterozygotic type	Aa**a**
D25	2n = 3x = 51	0.32 ± 0.0170	Red heterozygotic type	Aa**a**
D27	2n = 3x = 51	0.29 ± 0.0367	Red heterozygotic type	Aa**a**
FZ_3x	2n = 3x = 51	0.36 ± 0.0058	Red heterozygotic type	Aa**a**
GF_3x	2n = 3x = 51	0.00 ± 0.0005	White homozygotic type	a**a**a
Q11	2n = 3x = 51	0.00 ± 0.0006	White homozygotic type	a**a**a
Q27	2n = 3x = 51	0.00 ± 0.0002	White homozygotic type	a**a**a
B431	2n = 4x = 68	0.26 ± 0.0121	Red heterozygotic type	Aa**a**a
B432	2n = 4x = 68	0.27 ± 0.0165	Red heterozygotic type	Aaaa
B479	2n = 4x = 68	0.76 ± 0.0186	Red heterozygotic type	AAAa
B433	2n = 4x = 68	0.78 ± 0.0199	Red heterozygotic type	AAAa
B456	2n = 4x = 68	0.58 ± 0.0137	Red heterozygotic type	AAaa
H424	2n = 4x = 68	1.03 ± 0.0248	Red homozygotic type	AAAA
K474	2n = 4x = 68	0.76 ± 0.0149	Red heterozygotic type	AAAa
2008 No.3	2n = 4x = 68	0.57 ± 0.0121	Red heterozygotic type	AAaa

In the same series materials, the results of different ploidies lines were also consistent with each other. The B series was derived from ‘LQ1’, the diploid lines(varieties) included the DY lines, ‘JH1’ and H30-6, while the triploid line was B356, and the tetraploid lines were B431, B432, B433, B456, and B479. These lines (varieties) were all heterozygote except for ‘LQ1’, ‘DY2’ and ‘DY3’, which were AA homozygotes, indicating that these varieties (lines) received pollen carrying the white-fleshed recessive allele a when they were receptive to pollination. ‘SW’ (2x, Aa) and SW_3x (3x, AAa), ‘XZ’ (2x, Aa) and XZ_3x (3x, AAa), B356 (3x, Aaa) and B456 (4x, AAaa), and K374 (3x, AAa) and K474 (4x, AAAa) were generated from the same material, and the only difference between them was the difference in ploidy. The genotyping results showed that the proportion of allele A increased with increasing ploidy, which may be an important factor contributing to the difficulty of obtaining white-fleshed tetraploid materials.

### Ploidy identification in F_1_ progenies (4x × 2x and 2x × 4x)

The ploidy of F_1_ true hybrids was identified by using flow cytometry. The results showed that the hybrid ploidy was mainly the intermediate to the ploidies of the parents (i.e., triploid), but there were multiple types of ploidy variation (Table 2). When the female parent was tetraploid, the ploidy variation in the progenies was lower. The proportions of triploids in the progenies of B431 × ‘GF’, B456 × ‘HB1’ and H424 × ‘HB1’ were 98.04%, 94.12%, and 100.00%, respectively. B432 × ‘BTZ’ showed a slightly lower proportion of 83.63%. When the male parent was tetraploid, the proportion of triploids in the progenies was only approximately 50%. The reason may be that there is less abnormal behavior of chromosomes during the meiosis of female gametes than that of male gametes; similar results have been obtained in citrus [20]. In general, the main types of ploidy variation observed in the hybrids between tetraploid and diploid were diploid and tetraploid. It is speculated that fertile n gametes and 3n gametes produced by tetraploids were combined with diploid n gametes in these cases. One peculiar finding was that three lines of pentaploid materials appeared among the B456 × ‘HB1’ progeny.

**Table 2:** Ploidy identification results of loquat F_1_ hybrids

Type	Combination	Diploid (%)	Triploid (%)	Tetraploid (%)	Pentaploid (%)
4x × 2x	B431 × ‘GF’	1 (1.96%)	50 (98.04%)	/	/
	B432 × ‘BTZ’	7 (12.72%)	46 (83.63%)	2 (3.63%)	/
	B456 × ‘HB1’	/	80 (94.12%)	2 (2.35%)	3 (3.53%)
	H424 × ‘HB1’	/	5 (100.00%)	/	/
2x × 4x	‘GF’ × B431	9 (42.00%)	10 (48.00%)	2 (9.52%)	/
	‘BTZ’ × B432	5 (23.81%)	15 (71.43%)	1 (4.76%)	/
	‘HB1’ × B456	3 (17.65%)	10 (58.82%)	4 (23.53%)	/
	‘HB1’ × H424	1 (10.00%)	4 (40.00%)	5 (50.00%)	/

### Analysis of flesh color inheritance in 4x × 2x and 2x × 4x F_1_ hybrids

The combination of two flesh color markers was used to identify the true hybrids’ flesh color, and the inheritance of flesh color in polyploid loquats was analyzed (Table 3). According to the chi-square test results, all cross combinations included in this experiment showed values of less than Χ^2^ = 3.84, P = 0.05. This result indicated that the segregation of flesh color in tetraploids was conformed to Mendel’s classic genetics law, but there were slight differences due to the differences in the parent materials.

**Table 3:** Flesh color genotype segregation results of the hybrids from the crosses between diploid and tetraploid loquats

Cross	Ploidy	Flesh color type		Segregation ratio (R:W)	Expected ratio (R:W)	Chi-square	*P* value
		Red	White				
‘GF’ × B431	2x	4	5	1:1.25	1:3	1.81	0.18
(aa × Aaaa)	3x	5	5	1:1	1:1	0.00	1
	4x	2	0	1:0	3:1	0.67	0.42
	Total	11	10	1.1:11	1:1.38	0.92	0.37
B431 × ‘GF’	2x	0	1	0:1	1:3	0.33	0.57
(Aaaa × aa)	3x	20	30	1:1.5	1:1	2.00	0.16
	4x	0	0	/	/	/	/
	Total	20	31	1:1.55	1:1.02	2.16	0.16
‘BTZ’ × B432	2x	3	2	1.5:1	1:3	3.27	0.07
(aa × Aaaa)	3x	8	7	1:1.14	1:1	0.07	0.79
	4x	0	1	0:1	3:1	3.00	0.18
	Total	11	10	1:1.09	1:1.21	0.43	0.33
B432 × ‘BTZ’	2x	3	4	1:1.33	1:3	1.37	0.25
(Aaaa × aa)	3x	27	19	1.42:1	1:1	1.44	0.23
	4x	1	1	1:1	3:1	0.67	0.42
	Total	31	24	1.29:1	1:1.13	2.31	0.24
‘HB1’ × B456	2x	2	1	2:1	1:1	0.33	0.57
(aa × AAaa)	3x	10	0	1:0	5:1	2.00	0.15
	4x	3	1	3:1	1:1	1.00	0.32
	Total	15	2	7.5:1	2.28:1	0.91	0.23
B456 × ‘HB1’	2x	0	0	/	/	/	/
(AAaa × aa)	3x	66	14	4.71:1	5:1	0.04	0.84
	4x	1	1	1:1	1:1	0.00	1
	5x	3	0	1:0	1:0	0.00	1
	Total	70	15	4.73:1	3.77:1	0.57	0.84
‘HB1’ × H424	2x	1	0	1:0	1:0	0.00	1
(aa × AAAA)	3x	4	0	1:0	1:0	0.00	1
	4x	5	0	1:0	1:0	0.00	1
	Total	10	0	1:0	1:0	0.00	1
H424 × ‘HB1’	2x	0	0	/	/	/	/
(AAAA × aa)	3x	5	0	1:0	1:0	0.00	1
	4x	0	0	/	/	/	/
	Total	5	0	1:0	1:0	0.00	1

**Fig. 7:**
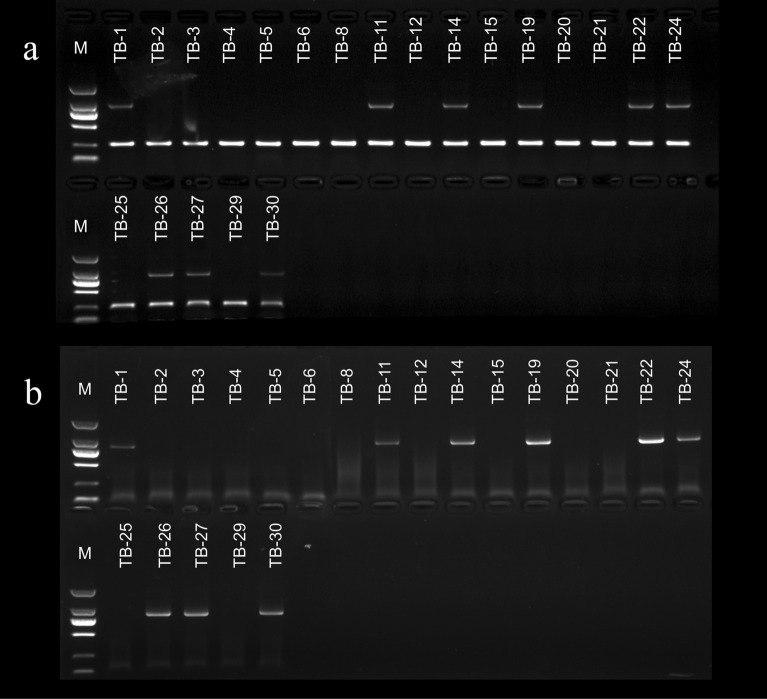
‘BTZ’ × B432 (TB hybridization) true hybrid flesh color-specific marker identification results. **a** Original marker. **b** Improved marker. The marker is the DL 2000 DNA marker

Figure 7 shows the flesh color identification results for 16 true hybrids from the aa × Aaaa cross combination of ‘BTZ’ × B432. The marker results identified two types of loquats: red-fleshed heterozygotes and white-fleshed homozygotes. This indicated that the flesh color of the hybrid progenies had segregated. The statistical results showed that the red- and white-fleshed segregation ratio of the triploid hybrids was 1:1.14 and that of all hybrids was 1:1.10. The chi-square test results showed conformation to Mendel’s law. The red- and white-fleshed segregation ratio of the triploid hybrids in the reverse cross combination, B432 × ‘BTZ’, was 1.42:1, that of all hybrids was 1.29:1. The chi-square test results showed conformation to Mendel’s law. However, the Χ^2^ value was too large, indicating that some deviation occurred under the conditions of Mendel’s law, mainly because the number of red-fleshed hybrids was larger than the expected value. The segregation ratio of the triploid hybrids in the same genotype combination, ‘GF’ × B431, was 1:1, and that of the triploid hybrids for B431 × ‘GF’ was 1:1.5. The chi-square test results conformed to Mendel's law, but the number of white-fleshed hybrids of B431 × ‘GF’ was larger than the expected value. The segregation ratio of the triploid hybrids in the AAaa × aa cross combination of B456 × ‘HB1’ was 4.71:1, which was close to the expected value. The three pentaploid hybrids were all red-fleshed heterozygotes, which may be produced from the tetraploid 4n gamete AAaa and the diploid n gamete a. The segregation ratio of the triploid hybrids in B456 × ‘HB1’ was 1:0, which was quite different from the expected value and tended to produce red-fleshed types. The hybrids of AAAA × aa cross combination H424 × ‘HB1’ and ‘HB1’ × H424 were both red-fleshed heterozygotes, and no trait segregation occurred.

## Discussion

### The qPCR genotyping system is an effective method for quick and convenient polyploid genotyping

The polyploid qPCR genotyping system proposed in this study can be used to quickly and easily estimate the allele dose of polyploid loquats and determine their genotypes. The parameter b showed a good clustering relationship in both triploids and tetraploids, and the correlation coefficient reached 0.9992. This method could significantly separate the heterozygous triploids and tetraploids, and the results conformed to expectations and exhibited high stability. The genotype of B431 (Aaaa) and B456 (AAaa) was only slightly different, but the parameter b results could be clearly divided into two types (Table 1). The simulated DNA pool results also showed that this method still presents high application prospects for accurately detecting the heterozygous genotypes of higher-ploidy materials.

There are several methods for estimating polyploid allele dose, among which KASPar technology [39, 43] and QF-PCR [44, 45] are widely applicable. KASPar technology is similar to TaqMan technology, and this method requires a high-density SNP database and genome-wide association analysis [46]. Therefore, KASPar technology is affected by the cost and research depth in the range of small plant applications [47], and the development of SNP chips is also more difficult for polyploids [48, 49]. QF-PCR encounters difficulty in the performance of quantitative analysis in polyploids, and the experimental procedure requires lid opening for manual dilution, which greatly increases the possibility of interference and contamination [38]. The qPCR genotyping system uses PCR amplification to amplify the initial allele copy number in the material into a fluorescent signal and detects the signal without lid opening during the process. The fluorescent dye employed in this system is SYBR Green I, which is widely employed, low cost, and simple to operate.

qPCR genotyping is more suitable for the genotyping of functional genes for important traits. It is not limited by the type of markers selected, and the procedure is simple. The qPCR genotyping primer design strategy focuses on large deletions, indel insertion/deletion sites or SNP sites of alleles. Meanwhile, mismatched bases can be introduced at the 3′ ends of the primers to improve their resolution [50, 51].

### The application of qPCR technology in polyploid allele-specific expression analysis and dose–effect research

After WGD occurs in plants, a large number of repetitive genes are produced. These repetitive genes have three different expression patterns, including expression level advantage, homology expression bias, and homology expression silencing [52]. These phenomena have been reported in species such as cotton [53], wheat [54] and *Brassica napus* [55]. Homologous expression deviation is when a locus has two alleles whose relative expression ratio may not match the relative copy number ratio [56]. For diploids, the expression of alleles can be determined by measuring the expression of alleles or by RNA-seq analysis to determine whether biased expression occurs [43, 57]. Polyploid alleles cannot be directly judged as occurring at a 1:1 ratio because of multiple heterozygotes [27].

WGD changes the regulation of gene expression, which involves complex dose effects and dose compensation effects [52, 58]. Plant ploidy does not directly correspond to the change trend of gene expression, which exist a kind of heterosis [59]. Liu et al. [60] found that the heterosis of triploid loquat may be caused by extensive genetic variation during the formation of triploids and changes in gene expression patterns. However, these studies often ignore inherent differences in gene copy numbers and cannot distinguish the specific genomic elements that cause changes in gene expression, such as gene copy numbers, promoters, gene bodies, and transposons [61, 62]. Whether there are differences in the phenotypes of different heterozygotes in polyploids and the reasons for the differences are research topics of considerable interest. qPCR can be used to quickly and accurately determine the relative copy number of polyploid alleles, which not only provides a method for studying the specific expression of polyploid alleles and gene dosage effects but can also be combined with RNA-seq and DNA methylation profiles to verify the results of each method.

### The application of qPCR genotyping in polyploid breeding and cross breeding

Most plants have experienced or are developing polyploidy [63]. The widespread existence of polyploids indicates that polyploids present higher selective advantages than diploids, such as a stronger colonization ability, higher tolerance, and greater ecological adaptability [64–66]. Polyploidy can be achieved by colchicine induction, seed selection, somatic fusion and hybridization [67, 68]. Common fruit trees have triploid varieties or germplasm resources, such as banana [69], citrus [70], apple [71], grape [72], loquat [19], and Chinese kiwi [73]. Somatic fusion can overcome conventional sexual reproduction obstacles and introduce interspecific or intergenus genomes to increase the diversity of breeding materials; however, among fruit trees, this has been successfully achieved only in citrus [74, 75]. Hybrid breeding is still the first choice for achieving high-quality polyploidy. Clear genotypes for important traits and the inheritance of polyploids are beneficial for improving the utilization rate of polyploid germplasm resources, the selection of excellent cross combinations and the analysis of marker- trait associations. The qPCR polyploid genotyping system presents broad application prospects for the identification of unknown germplasm resources, parent selection and early progeny selection.

In this study, the triploid ratio for a given parent material was higher when it was used as the female parent than as the male parent, and F_1_ triploids accounted for an average of 93.94% in the progenies, which was consistent with Liang's results [76]. Similar results have been found in citrus [20], *Hydrangea* [77]. The cross between tetraploid and diploid is an effective method for rapidly obtaining a large number of triploids, which present heterosis and selective advantages [11, 19, 20]. In general, it is more difficult to perform a cross when tetraploids are used as male parents because they obtain fewer seeds and a low seedling rate. Considering the cost of maintenance, tetraploids should be given priority as female parents.

### The effect of qPCR genotyping for polyploid flesh color on the genetic improvement of loquat

Polyploid loquats present higher application value whether they are directly cultivated or used as a rootstock [19, 78]. Triploid loquat breeding is one of the most effective methods of breeding seedless loquats [18, 79]. Most of the existing polyploid loquats were obtained from seed selection with complex sources, making the genotyping of important economic traits necessary. White-fleshed loquats are the main direction of the development of loquat breeding in the future [22, 25]. The lack of white-fleshed tetraploid parents has prevented rapid, large-scale variety breeding.

In this study, a qPCR polyploid genotyping system was used to determine loquat flesh color genotypes, and it was clear that tetraploid loquats from different sources have different heterozygous genotypes. Under the premise of Mendel's law, the flesh color separation ratio of the F_1_ hybrids has a slight deviation, which may be caused by a variety of factors, and the specific reasons need to be further studied. Two tetraploid loquats, B431 and B432, with the Aaaa genotype, can be used as excellent parents for breeding white-fleshed polyploid loquats. The proportion of white-fleshed hybrids obtained from B431 as the female parent was much greater than that form B432, which may have been due to the different abnormalities in meiosis in the two materials. Liang et al. [76] observed B-series tetraploid meiosis and showed that the chromosomal configuration of diakinesis in B431 was complex and that the proportion of bivalents and tetravalents was lower than in other homologous materials. Loquat has a narrow genetic background, and natural tetraploids are largely autotetraploid. The frequency of tetravalents during meiosis in B-series tetraploid loquat is approximately 20% [76]. In the preferential pairing of homologous chromosomes, the transmission of parental heterozygosity decreases as the tetrasomy rate (τ) increases [80]. Garavello et al. [45] found that the Parental heterozygosity restitution (PHR) of tetraploid ‘Moncada’ female gametes was higher and that the progenies of tetraploid female parents presented higher genotype consistency with the parent. Accurate assessment of preferential pairing and multivalent formation in tetraploids can accelerate the accumulation of rare but advantageous alleles through MAS [46, 48].

## Conclusions

This study shows that qPCR technology can be used for polyploid genotyping and can effectively distinguish different heterozygotes. The method presents the advantages of accurate results, simple operation, rapid detection and low cost. This technology has been successfully applied for flesh color genotyping in polyploid loquat to clarify the flesh color genotypes of the existing representative polyploid materials and improve the germplasm utilization efficiency. It provides a method for studying population inheritance, dose effect and allele-specific expression. In addition, the gene segregation of tetraploid flesh color conforms to Mendel's law, but there are slight differences among different materials. This provides a theoretical basis for breeding white-fleshed triploid varieties by using red-fleshed-heterozygous tetraploids as parents, among which tetraploid B431 and B432 can be used as the backbone parents of hybrids. The qPCR genotyping and MAS model can be used as a breeding system for quickly screening target parents to obtain excellent cross combinations and as a reference strategy for the genetic improvement of loquat and other species.

## Methods

### Plant materials

In this experiment, 8 red-fleshed tetraploid loquat lines, 14 red-/white-fleshed triploid loquat lines (varieties), and 27 red-/white-fleshed diploid loquat lines (varieties) were collected from the Loquat Germplasm Garden (Table 4), College of Horticulture and Landscape Architecture, Southwest University, Chongqing, China. In order to explore the flesh color segregation rules of progenies of tetraploid loquats with different genotypes, according to our qPCR genotyping results, tetraploid loquats of different genotypes were crossed with white-fleshed diploid loquats (Table 5). The parents were emasculated, artificially pollinated, and bagged in November of the first year, and seeds were collected and sown in April of the following year when the fruit was mature. The F_1_ hybrids were used to study ploidy segregation and the inheritance of flesh color.

**Table 4:** Forty-nine loquat lines (varieties) used for this study

Lines (varieties)	Abbreviation	Ploidy	Flesh color
B431	B431	2n = 4x = 68	Red
B432	B432	2n = 4x = 68	Red
B433	B433	2n = 4x = 68	Red
B456	B456	2n = 4x = 68	Red
B479	B479	2n = 4x = 68	Red
H424	H424	2n = 4x = 68	Red
K474	K474	2n = 4x = 68	Red
2008 No. 3	20,083	2n = 4x = 68	Red
A313	A313	2n = 3x = 51	Red
A322	A322	2n = 3x = 51	Red
B356	B356	2n = 3x = 51	Red
D6	D6	2n = 3x = 51	Red
D25	D25	2n = 3x = 51	Red
D27	D27	2n = 3x = 51	Red
K374	K374	2n = 3x = 51	Red
Sengweizaoseng (3x)	SWZS_3x	2n = 3x = 51	Red
Donghuzao (3x)	DHZ_3x	2n = 3x = 51	Red
Xiangzhong (3x)	XZ_3x	2n = 3x = 51	Red
Fuzao (3x)	FZ_3x	2n = 3x = 51	Red
Guifei (3x)	GF_3x	2n = 3x = 51	White
Q11	Q11	2n = 3x = 51	White
Q27	Q27	2n = 3x = 51	White
‘Longquan No. 1’	LQ1	2n = 2x = 34	Red
‘Danyou No. 1’	DY1	2n = 2x = 34	Red
‘Danyou No. 2’	DY2	2n = 2x = 34	Red
‘Danyou No. 3’	DY3	2n = 2x = 34	Red
‘Jinhua No.1’	JH1	2n = 2x = 34	Red
‘Senweizaosheng’	SWZS	2n = 2x = 34	Red
‘Zaozhong No. 6’	ZZ6	2n = 2x = 34	Red
‘Hunanzaoshu’	HNZS	2n = 2x = 34	Red
‘Xingning No. 1’	XN1	2n = 2x = 34	Red
‘Donghuzao’	DHZ	2n = 2x = 34	Red
‘Xiangzhong’	XZ	2n = 2x = 34	Red
‘Marc’	Marc	2n = 2x = 34	Red
‘Pelluches’	Pell	2n = 2x = 34	Red
H30-6	H30-6	2n = 2x = 34	Red
Changbai No. 1	CB1	2n = 2x = 34	White
Guanyu	GY	2n = 2x = 34	White
Q14	Q14	2n = 2x = 34	White
‘Dayangwanbai’	DYWB	2n = 2x = 34	White
‘Ninghaibai’	NHB	2n = 2x = 34	White
‘Ruantiaobaisha’	RTBS	2n = 2x = 34	White
‘Baili’	BL	2n = 2x = 34	White
‘Baiyu’	BY	2n = 2x = 34	White
Spain white	SW	2n = 2x = 34	White
Guiye white	GW	2n = 2x = 34	White
‘Huabai No. 1’	HB1	2n = 2x = 34	White
‘Guifei’	GF	2n = 2x = 34	White
‘Bingtangzhong’	BTZ	2n = 2x = 34	White

**Table 5:** cross combination of loquat polyploid

Type	Cross	Abbreviation
4x × 2x	B431 × ‘GF’	BG hybridization
	B432 × ‘BTZ’	BT hybridization
	B456 × ‘HB1’	BH hybridization
	H424 × ‘HB1’	H4H hybridization
2x × 4x	‘GF’ × B431	GB hybridization
	‘BTZ’ × B432	TB hybridization
	‘HB1’ × B456	HB hybridization
	‘HB1’ × H424	HH4 hybridization

### Extraction of genomic DNA

Loquat leaflets of 49 lines (varieties) and F_1_ hybrids were collected and brought back to the laboratory, where they were stored in the refrigerator at − 80 °C. The genomic DNA (gDNA) extraction method referred to Wen Guo's improved CTAB method [42]. DNA quality was detected by 1.0% agarose gel electrophoresis, and high-quality gDNA was diluted to 50 ng/μL for PCR and qPCR analyses.

### Identification and optimization of specific molecular markers for loquat flesh color

Flesh color-specific molecular marker obtained from Fu et al*.* [25] and Zou et al*.* [23] was used to preliminarily identify the lines (varieties). The flesh color-specific marker amplification system and PCR program were run according to the literature with slight modifications [23]. The sequences of the marker primers are shown in Table S1. The electrophoretic map contains one *EjPSY2A* band and one *EjPSY2A*^*d*^ band. The length of the two bands of the original marker is quite different, and the long band cannot be observed in some materials. Errors are prone to occur when using this marker to screen unknown materials. Therefore, according to the analysis results for the *EjPSY* sequence, a different reverse primer was designed for specific markers (Additional file 4: Table S1). Only the 872 bp product was obtained for *EjPSY2A*, and materials with a known genotype were used for marker verification.

### Design of the qPCR genotyping system

Flesh color genotyping was carried out by qPCR with gDNA (analytikjena qTOWER^3^), and a DNA sequence with a constant copy number in the genome was used as an internal control. The SSR marker CH03g12 shows no polymorphism in loquat, and its copy number only changes with ploidy [42]. CH03g12, *actin* [60] and *H4-1* were used for the screening of loquats with different ploidies to identify primers with high consistency for generating the reference DNA sequence. According to the sequences of *EjPSY2A* (allele A) and *EjPSY2A*^*d*^ (allele a), the allele A-specific primer q2A and the alleles A and a specific primer pair q2A/2Ad were designed (Additional file 1: Fig. S1). The qPCR system included gDNA (2 μL), primers (0.2 μL), ddH2O (3.6 μL), and 2 × NovoStart^®^ SYBR qPCR Super Mix Plus (Novoprotein Scientific Inc.) (5 μL), the reaction program was 95 °C for 1 min, followed by 40 cycles (95 ℃ for 20 s, 60 ℃ for 1 min), and then melting curve analysis at 60–95 ℃.

A mixed white-fleshed diploid pool (W-mix) was prepared from ‘HB1’, ‘BTZ’ and ‘GF’, and a mixed red-fleshed homozygous diploid pool (R-mix) was prepared from ‘LQ1’, ‘DY2’ and ‘DY3’. The DNA concentration in the pool was 50 ng/μL, and the two pools were mixed at ratios of 9:1, 5:1, 3:1, 2:1, 3:2, 1:1, 2:3, 1:2, 1:3, 1:5 and 1:9 to construct the simulated heterozygote library with different proportions of allele A [39]. The 2:1 and 1:2 ratios simulated triploid heterozygotes, and the 3:1, 1:1, and 1:3 ratios simulated tetraploid heterozygotes. The mixtures were used together with two DNA pools to test the ability of the qPCR genotyping strategy to distinguish heterozygotes in triploids and tetraploids.

### qPCR genotyping data analysis

The relative copy numbers of the target gene were calculated according to the 2^−ΔΔCt^ method. If the control was a heterozygous diploid, the relative copy number of allele A was a_1_, the relative copy number of allele A and a was a_3_, and the relative copy number of allele a was a_2_ (a_2_ = a_3_ – a_1_). From the a_1_ and a_2_ normalized values, the relative A allele signal [b = a_1_/(a_1_ + a_2_); 0 ≤ b ≤ 1] of each simulated sample were calculated. The genotypes of materials with different ploidies and different relative copy numbers were calculated as follows:

$${\text{Tetraploid}}:{ 2} \times {\text{b}},{\text{ AAAA}};{ 3} \times {\text{b}}/{2},{\text{ AAAa}};{\text{ b}},{\text{ AAaa}};{\text{ b}}/{2},{\text{ Aaaa}}; \, 0,{\text{ aaaa}}.$$

$${\text{Triploid}}:{ 2} \times {\text{b}},{\text{ AAA}};{ 4} \times {\text{b}}/{3},{\text{ AAa}};{ 2} \times {\text{b}}/{3},{\text{ Aaa}}; \, 0,{\text{ aaa}}.$$

The theta angle [θ = tan^−1^(a_2_/a_1_); 0º ≤ θ ≤ 90º] of each simulated sample was also calculated for analysis.

### qPCR genotyping of flesh color in polyploid loquats

**Fig. 8:**
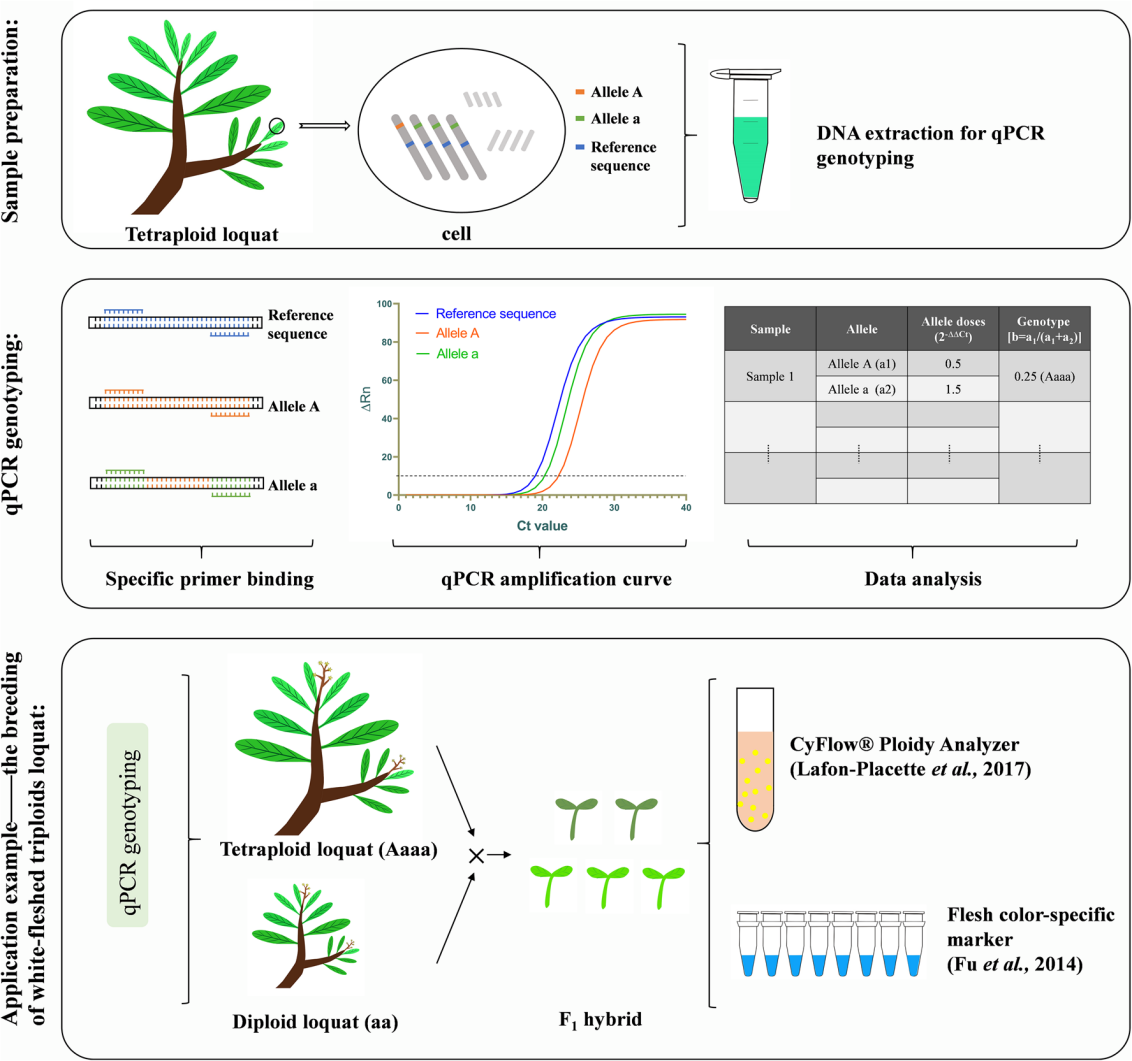
Schematic flew diagram of qPCR genotyping. DNA of loquat leaves was extracted for qPCR genotyping. qPCR genotyping was amplified by allele-specific primers, and the relative copy number of each allele was calculated by 2^−ΔΔCt^ method, and then parameter b [b = a_1_/(a_1_ + a_2_); 0 ≤ b ≤ 1] was calculated to determine the genotype of the tested material. We can select tetraploids of the appropriate genotype as backbone parents according to the breeding goal. More genetic information was obtained by MAS and ploidy analysis

The diploid loquats were genotyped by qPCR, and the results were calibrated with the results of the color-specific molecular marker analyses to verify the effectiveness of the qPCR genotyping system. The triploid and tetraploid loquats were genotyped by qPCR, and the flesh color genotypes of the triploid and tetraploid loquats were determined. A flow diagram of the experimental operation is illustrated in Fig. 8.

### Hybrid identification, ploidy identification and flesh color-specific marker identification in the F_1_ hybrids

The F_1_ hybrids were identified by hybrid identification, ploidy identification, and flesh color-specific marker analysis; the segregation ratios of red-fleshed and white-fleshed loquats were calculated; and the inheritance of tetraploid flesh color was explored. SSR markers were used to identify true and false hybrids. The SSR markers were derived from loquat genome data (Additional file 4: Table S2). For each cross combination, three SSR markers on different chromosomes were used. When two or more male parent-specific bands appeared in the F_1_ hybrids, they were considered to be true hybrids. The PCR amplification and electrophoresis of SSRs were run according to the literature with slight modifications [42, 81].

The ploidy of hybrids was identified by flow cytometry [82]. Fresh young plant tissue (1 g) was placed in a culture dish, and 1 ml of nuclear extract was added after chopping. The liquid was filtered into a 2 mL centrifuge tube through a 30 μm filter membrane and centrifuged at 1000 r/min for 5 min. The supernatant was discarded, 500 μL of cell nucleus extract was added to prepare a cell nucleus suspension, 50 μL of DAPI (5 μg/mL) was added, and the cells were stained for 3–4 min in the dark and then tested on a CyFlow^®^ Ploidy Analyzer (Sysmex-Partec GmbH).

## Supplementary Information

**Additional file 1:Fig. S1**. Schematic diagram of qPCR genotyping primer positions.

**Additional file 2**: **Fig. S2**. The qPCR amplification curves and melting curve for Cho3g12, q2A, q2A/2Ad.

**Additional file 3:Fig. S3**. Map of *EjPSY2A*^*d*^ chromosome localization and different tetraploid heterozygous genotypes.

**Additional file 4:Table S1**. Primer of flesh color specific molecular marker and qPCR genotyping. **Table S2**. SSR marker sequence for hybrid identification.

## Data Availability

The datasets supporting the conclusions and a description of the complete protocol are included within the article.

## Abbreviations

WGD: Whole-genome duplication
MAS: Marker-assisted selection
Ct: Cycle threshold
GBS: Genotyping by sequencing
QF-PCR: Quantitative fluorescent polymerase chain reaction
HRM: High-resolution melting
qPCR: Quantitative real-time polymerase chain reaction
gDNA: Genomic DNA
SSR: Simple sequence repeat
SNP: Single nucleotide polymorphism
