# Supplementary material for: Genotyping of polyploid plants using quantitative PCR: application in the breeding of white-fleshed triploid loquats (Eriobotrya japonica)
Source: Plant Methods. 2021 Sep 3;17:93. doi: 10.1186/s13007-021-00792-9 (PMC8418031; doi:10.1186/s13007-021-00792-9)
Supplement: Supplementary file 1 — Additional file 1:Fig. S1. Schematic diagram of qPCR genotyping primer positions. [file 13007_2021_792_MOESM1_ESM.docx]

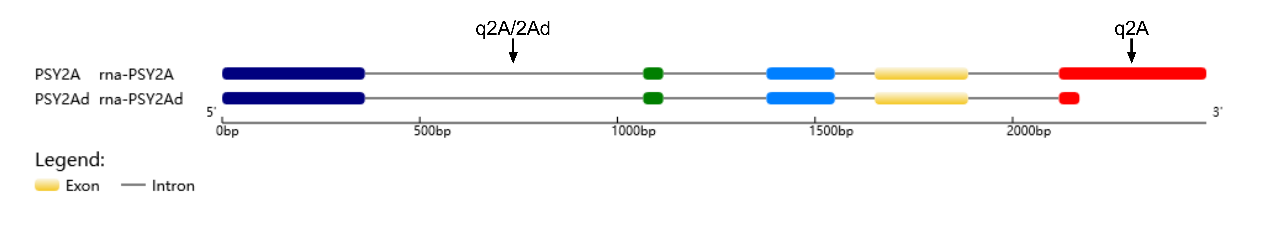


**Fig. S1 Schematic diagram of qPCR genotyping primer positions.** *EjPSY2A* and *EjPSY2A^d^* are a pair of alleles in loquat. They control the color formation of loquat flesh. *EjPSY2A* is the dominant gene of red flesh (denoted as A), *EjPSY2A^d^* is the recessive gene of white flesh (denoted as a). Red-fleshed genotype is AA or Aa, white-fleshed genotype is aa. Partial deletion of EjPSY2Ad in white-fleshed loquat results in functional defects relative to red-fleshed loquat According to the sequence of *EjPSY2A* and *EjPSY2A^d^*, design allele A specific primer q2A, allele A and a specific primer q2A/2Ad.
