# Supplementary material for: Genotyping of polyploid plants using quantitative PCR: application in the breeding of white-fleshed triploid loquats (Eriobotrya japonica)
Source: Plant Methods. 2021 Sep 3;17:93. doi: 10.1186/s13007-021-00792-9 (PMC8418031; doi:10.1186/s13007-021-00792-9)
Supplement: Supplementary file 2 — Additional file 2: Fig. S2. The qPCR amplification curves and melting curve for Cho3g12, q2A, q2A/2Ad. [file 13007_2021_792_MOESM2_ESM.docx]

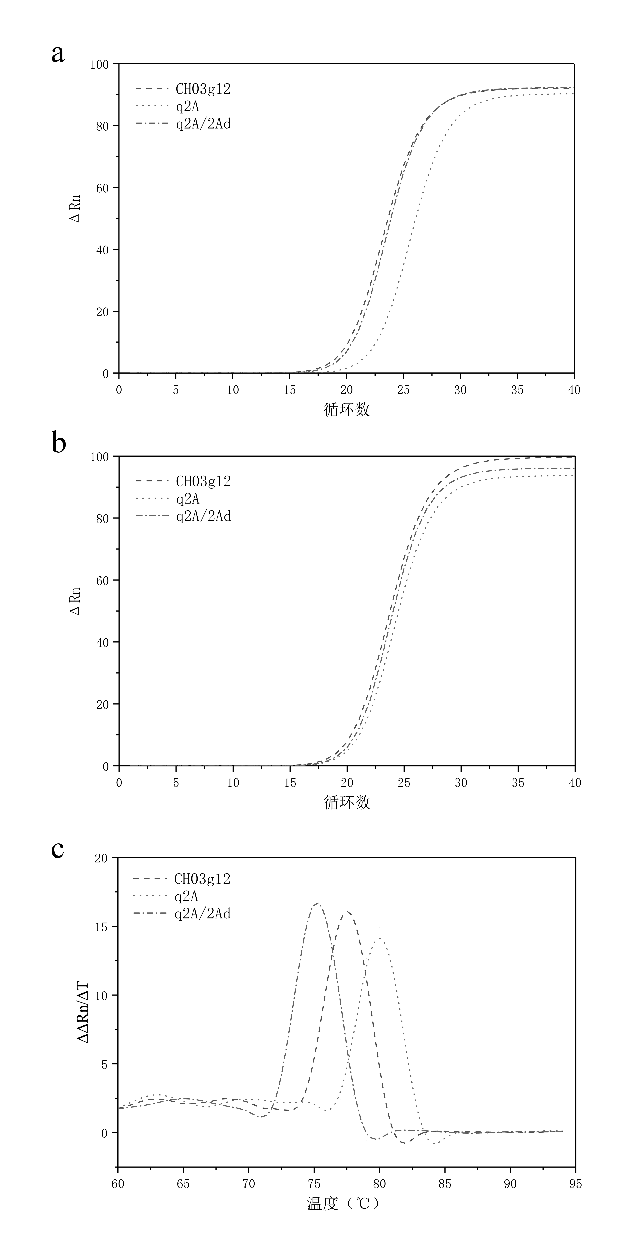


**Fig. S2 The qPCR melting curves and melting curve for Cho3g12, q2A, q2A/2Ad.** (a) B432 amplification curve; (b)B479 amplification curve; (c)Melting curve. q2A/2Ad is the specific primer of *EjPSY2A* and *EjPSY2A^d^*, q2A is the specific primer of *EjPSY2A*, Ch03g12 is the reference sequence. The genotype of tetraploid loquat B432 is Aaaa. The genotype of tetraploid loquat B479 is AAAa. The Ct values of the two lines were reasonable, and the amplification curves were obviously different. The specific primers q2A and q2A/2Ad have high specificity.
