# Supplementary material for: Genotyping of polyploid plants using quantitative PCR: application in the breeding of white-fleshed triploid loquats (Eriobotrya japonica)
Source: Plant Methods. 2021 Sep 3;17:93. doi: 10.1186/s13007-021-00792-9 (PMC8418031; doi:10.1186/s13007-021-00792-9)
Supplement: Supplementary file 3 — Additional file 3:Fig. S3. Map of EjPSY2Ad chromosome localization and different tetraploid heterozygous genotypes. [file 13007_2021_792_MOESM3_ESM.docx]

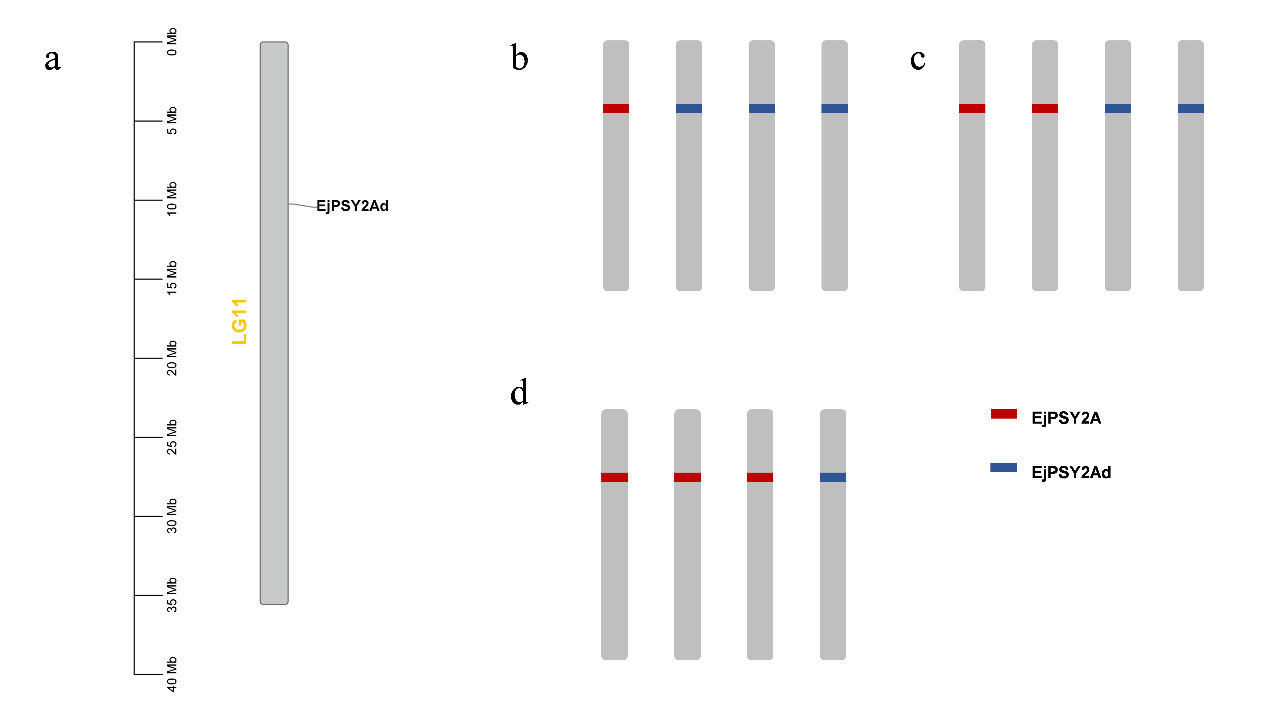


**Fig. S3 Map of** ***EjPSY2A^d^* chromosome localization and different tetraploid heterozygous genotypes.** (a) Map of *EjPSY2A^d^* chromosome localization; (b), (c) and (d) are tetraploid heterozygous genotypes of Aaaa, AAaa and AAAa, respectively. The *EjPSY2A* is the allele A, the *EjPSY2A^d^* is the allele a.
