# Supplementary material for: Genotyping of polyploid plants using quantitative PCR: application in the breeding of white-fleshed triploid loquats (Eriobotrya japonica)
Source: Plant Methods. 2021 Sep 3;17:93. doi: 10.1186/s13007-021-00792-9 (PMC8418031; doi:10.1186/s13007-021-00792-9)
Supplement: Supplementary file 4 — Additional file 4:Table S1. Primer of flesh color specific molecular marker and qPCR genotyping. Table S2. SSR marker sequence for hybrid identification. [file 13007_2021_792_MOESM4_ESM.docx]

Table S1. **Primer of flesh color specific molecular marker and qPCR genotyping**

| Primer | Primer sequences (5'→3') |
| --- | --- |
| EjPSY2A | F: TATGAACCATTGATTAGTCTAGC  R: GTTATTGTCACCGTAGTCGC |
| EjPSY2A-new | F: TATGAACCATTGATTAGTCTAGC  R: GCCACCATCATTCCAATC |
| CH03g12 | F: GCGCTGAAAAAGGTCAGTTT  R: CAAGGATGCGCATGTATTTG |
| actin | F: ATCCTTCGTCTGGACCTTGC  R: GACAATTTCCCGTTCAGCAGT |
| H4-1 | F: GCGAAACGACATCGTAAGGT  R: TGTACGTCACAGCATCACGA |
| q2A | F: GGTGGATATGAATGGAATGATGGT  R: GGCTAATGAGATCCGACGTTATTG |
| q2A/2Ad | F: TTTTTGCTGACTGTTTATCTGCTCA  R: AAACCACTTAAGACTCACGTGT |

Table S2. **SSR marker sequence for hybrid identification.**

| Combination | SSR Maker | Chromosome | Primer sequences (5'→3') |
| --- | --- | --- | --- |
| Guifei × B431 | MK12939 | Chr2 | F: CAAGGGTCTGTCCATTTCGT  R: GAAGTTCAACGATGGTGGTTT |
|  | MK103875 | Chr14 | F: ACAAAACAGTGGGTGGGAGA  R: GCACTGGAGCAAATTGAACA |
|  | MK89295 | Chr12 | F: ATAGCTGGAAAGTGGCATGG  R: CCTTCGAGCTCTCTCCCTTT |
| B431 × Guifei | MK26414 | Chr3 | F: AAGGGCAGAATCAAAGCGTA  R: AGGTTTACCCGGCAGAGAAT |
|  | MK 26820 | Chr3 | F: GAACCCGATTACCCGAAGAAG  R: GAGGAGGAGGAGGAGAAGA |
|  | MK 63685 | Chr8 | F: TGGGCTTATAGGTGAGAAAATG  R: TGGTGCTATGGCAACTCAAA |
| Bingtangzhong × B432 | MK 89295 | Chr12 | F: ATAGCTGGAAAGTGGCATGG  R: CCTTCGAGCTCTCTCCCTTT |
|  | MK 96623 | Chr13 | F: AAGGAGGGAGGGACACAAGT  R: CAGCCGTGGACAAGTCAATA |
|  | MK 116957 | Chr16 | F: GGACGCCAATTTGCATAAAC  R: CATCCACCCACCAATTCATA |
| B432 × Bingtangzhong | MK 24030 | Chr3 | F: GTCTTCGACGCTTCTTCAGG  R: GGGACAAGTTTAGCGGATCA |
|  | MK 105944 | Chr14 | F: GGAGGGGAAGAATGAGGAAG  R: AAGCATACAGCTTCGGGACT |
|  | MK 116957 | Chr16 | F: GGACGCCAATTTGCATAAAC  R: CATCCACCCACCAATTCATA |
| Huabai No.1 × H424 | MK 1495 | Chr1 | F: GTGCCACATTTGAAGCACAC  R: AGCACCCCAAAATTCTCATT |
|  | MK 26044 | Chr3 | F: TGTCCCTCACCATTCATTCA  R: GCTGTTTTCAGCCCTCTGAC |
|  | MK 116957 | Chr16 | F: GGACGCCAATTTGCATAAAC  R: CATCCACCCACCAATTCATA |
| H424 × Huabai No.1 | MK 11127 | Chr2 | F: GCAGTCCCAAGGAAATGCTA  R: CACCTCCGGACTACGATGTT |
|  | MK 26044 | Chr3 | F: TGTCCCTCACCATTCATTCA  R: GCTGTTTTCAGCCCTCTGAC |
|  | MK 116957 | Chr16 | F: GGACGCCAATTTGCATAAAC  R: CATCCACCCACCAATTCATA |
| Huabai No.1 × B456 | MK 1495 | Chr1 | F: GTGCCACATTTGAAGCACAC  R: AGCACCCCAAAATTCTCATT |
|  | MK 26044 | Chr3 | F: TGTCCCTCACCATTCATTCA  R: GCTGTTTTCAGCCCTCTGAC |
|  | MK 16957 | Chr16 | F: GGACGCCAATTTGCATAAAC  R: CATCCACCCACCAATTCATA |
| B456 × Huabai No.1 | MK 11127 | Chr2 | F: GCAGTCCCAAGGAAATGCTA  R: CACCTCCGGACTACGATGTT |
|  | MK 10237 | Chr2 | F: TTAATGGCGGGAGAGAAATG  R: AGAAGCAAGGCAAGGTACGA |
|  | MK 16957 | Chr16 | F: GGACGCCAATTTGCATAAAC  R: CATCCACCCACCAATTCATA |
